# Feasibility study and direct extraction of endogenous free metallic cations combining hemodialysis and chelating polymer

**DOI:** 10.1038/s41598-021-99462-y

**Published:** 2021-10-07

**Authors:** Marco Natuzzi, Coralie Grange, Thomas Gréa, Thomas Brichart, Axel Aigle, Denise Bechet, Benoit Hautefeuille, Eloise Thomas, Jean-Yves Ayoub, Jeanne-Marie Bonnet, Vanessa Louzier, Bernard Allaouchiche, Aymeric Couturier, Alexandra Montembault, Paula Nunes de Oliveira, Laurent David, François Lux, Olivier Tillement

**Affiliations:** 1 MexBrain, 13 Avenue Albert Einstein, 69100 Villeurbanne, France; 2 ILM, Institut Lumière Matière, UMR 5306, CNRS, Université Lyon1, Université de Lyon, Villeurbanne Cedex, France; 3 LAGEPP, Laboratoire d’Automatique, de Génie des Procédés et de Génie Pharmaceutique, UMR 5007, CNRS, Université Lyon1, Université de Lyon, Villeurbanne Cedex, France; 4 APCSe Agressions Pulmonaires et Circulatoires dans le Sepsis, VetAgro Sup, Université de Lyon, 69280 Marcy l’Etoile, France; 5 Service de Néphrologie et Dialyse, Assistance Publique-Hôpitaux de Paris (APHP), Hôpital Universitaire Ambroise Paré, Boulogne Billancourt, France; 6 IMP, Ingénierie Des Matériaux Polymères, UMR 5223, CNRS, Université Lyon1, Université de Lyon, Villeurbanne Cedex, France; 7 Institut Universitaire de France (IUF), 75231 Paris, France

**Keywords:** Biotechnology, Medical research, Chemistry

## Abstract

In this article, we report the conception and the use of dialysis-based medical device for the extraction of metals. The medical device is obtained by addition in the dialysate of a functionalized chitosan that can chelate endogenous metals like iron or copper. This water-soluble functionalized chitosan is obtained after controlled reacetylation and grafting of DOTAGA. Due to the high mass of chitosan, the polymer cannot cross through the membrane and the metals are trapped in the dialysate during hemodialysis. Copper extraction has been evaluated in vitro using an hemodialysis protocol. Feasibility study has been performed on healthy sheep showing no acute toxicity througout the entire dialysis procedure and first insights of metallic extraction even on healthy animals.

## Introduction

Metallic deregulation of endogenous metals and metal overloads are known for years to be tightly correlated with diseases like Wilson disease (copper)^1^ or hemochromatosis (iron)^2^. These genetic diseases are currently treated by chelation therapy or even simple phlebotomy when possible for iron^3,4^. Traditional chelation therapy is limited by secondary adverse effects, lack of compliance and even in some cases by neurological worsening for Wilson disease^5^. During last decades, metal dyshomeostasis and more subtle overloads of endogenous redox active metals like copper or iron have also been suspected to be correlated with pathologies where oxidative stress is recognized to have an important role^6^. Recent studies have deciphered the central role of iron for neurological disorders like Parkinson’s disease^7^ or Alzheimer’s disease^8^. These two proteinopathies would closely be linked to the presence of misfolded and aggregated proteins that have lost their physiological properties and acquired neurotoxic properties^9^. For these pathologies, metal ions are involved in initiation, acceleration and modification of aggregation pathways but also in neurodegeneration and associated oxidative stress^10^. In the last decade, iron chelation using deferiprone has been tested in two clinical trials (DeferipronPD:NCT01539837 and Fair-Park I: NCT00943748) to prevent dopaminergic neurons from neurodegeneration in Parkinson’s disease^11,12^. For these two clinical trials, a reduction of iron levels in specific regions of the brain was shown to be associated with an improvement of the motor functions (Phase 2 clinical trial Fair-Park II:NCT02655315 is ongoing).

Even more interestingly, important levels of unbound iron in plasma have been associated with lower vital prognosis for patients in intensive care units for different pathologies where free iron or copper can be released either by hemolysis and/or different cell deaths^13^. This is for example observed in sepsis, which is associated with particularly bad prognosis. Indeed, mortality of sepsis is estimated at 25.8% in intensive care units and 35.3% in hospitals^14^. In sepsis conditions, over-production of reactive oxygen species and reactive nitrogen species is observed both in the blood circulation and in damaged organs^15^. Elevated free iron and transferrin saturation in serum have been recently associated with an increased mortality in patients with sepsis in a retrospective study on 1891 patients^16^. A dose dependent increase in the risk was found as the iron level increase^17^. Impact of iron has also been emphasized in chronic liver diseases with higher iron levels for patients with alcoholic liver diseases, nonalcoholic fatty liver disease and hepatitis C viral infection^18^. For acute-on-chronic liver failure, higher incidence of multiorgans failure and consequent mortality is observed for patients with dysregulated iron homeostasis^19^. Kidney is another organ at risk for iron dysregulation^20^. Acute kidney injury is a relatively common disease associated with high mortality; it can be seen in 5–7% of hospitalized patients and in 57% of patients in intensive care units. Clinical studies have shown that higher plasma concentration of catalytic iron is correlated with significantly greater risk of deaths^21^. Level of catalytic iron is an important factor during cardiac surgery. After surgery, patients with higher levels display greater odds of acute kidney injury, mortality and postoperative myocardial injury^22^. Rhabdomyolysis, a muscular disorder, can also lead to acute kidney disease and in the most severe forms to death for a large fraction of patients^23^. During rhabdomyolysis, myoglobin is massively released from the muscle tissues and is directly associated with increase of oxidative stress^24^.

**Figure 1:**
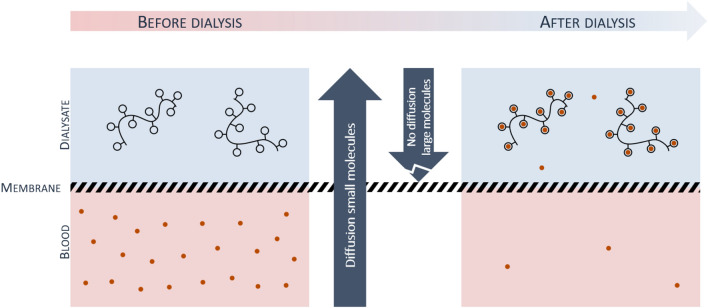
Principle of hemodialysis combined to chelating polymers for metallic extraction of the blood. During hemodialysis, chelating polymers cannot pass through the dialysis membrane in comparison with free metallic ions and metallic ions bound to small molecules. Metallic ions are then trapped by chelating polymers and are concentrated in dialysate.

All these examples emphasize the importance of extracting the excess of endogenous loosely bounds metals from blood in some patients. Even if chelating drugs are highly efficient to treat the majority of the metal overloads, in some specific cases, due to side effects or insufficient efficacy of chelating molecules, metal overloads can’t be treated adequately leading to life-threatening situations in particular for acute treatment in intensive care units where release of redox active metals is a critical parameter. The aim of this article is to propose the concept of a safe medical device capable of extracting excess of toxic endogenous metals from blood with a particular focus on copper for the treatment of Wilson disease. It is based on a simple combination of hemodialysis (HD) and chelating polymer solution placed in the dialysis fluid (Fig. 1).

**Figure 2:**
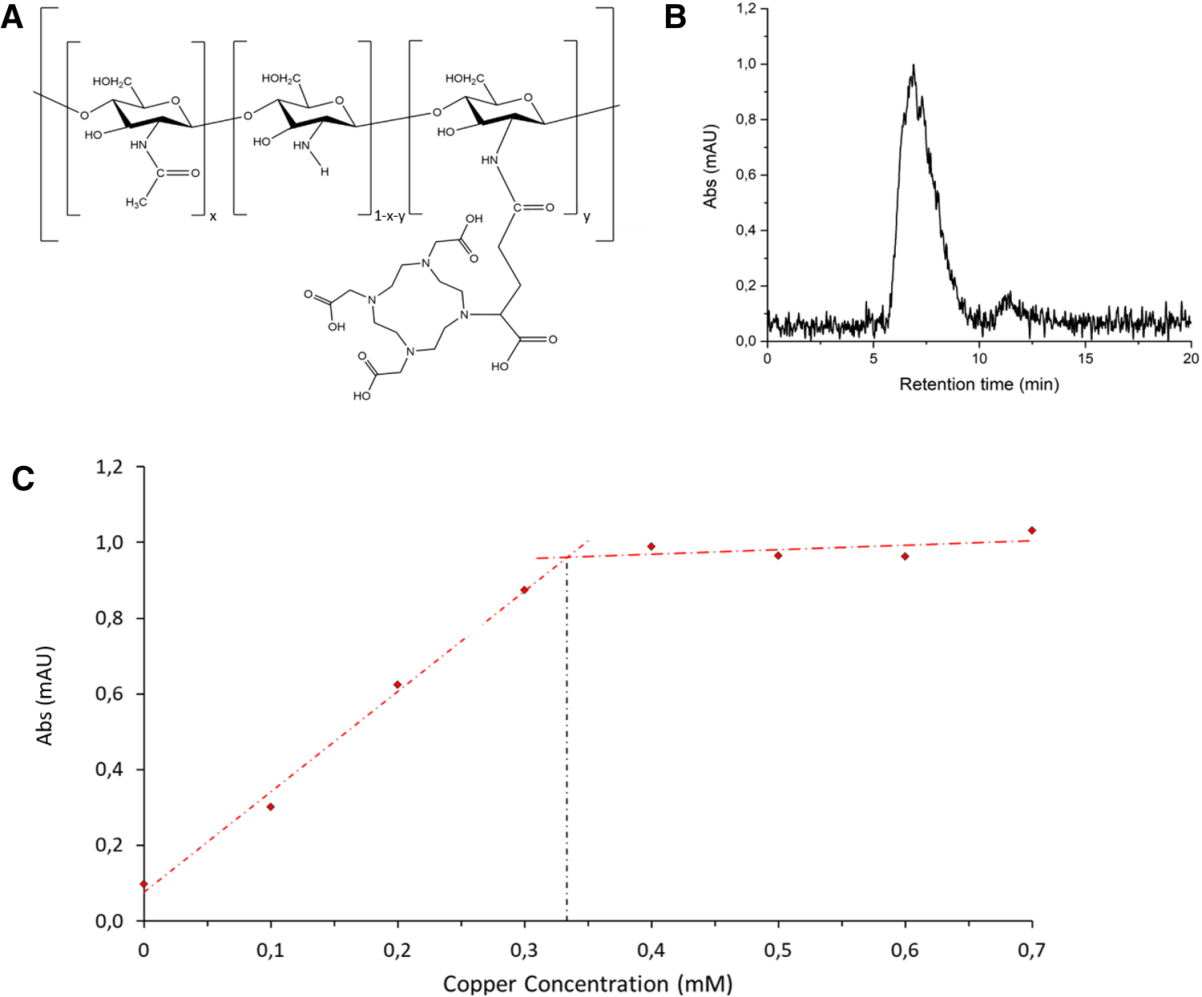
(**A**) Representation of reacetylated chitosan functionalized by DOTAGA: Chitosan@DOTAGA (x corresponds to acetylation ratio and y to DOTAGA grafted ratio). (**B**) SEC HPLC/UV elution diagram of Chitosan@DOTAGA in aqueous medium in absence of Cu(II) using detection at 295 nm (milli absorption units). (**C**) Dosage of a solution of 1 g L^−1^ of Chitosan@DOTAGA by Cu(II) using HPLC–UV SEC and plotting peak at 6.7 min.

The chelating polymer is a reacetylated chitosan functionalized by DOTAGA ligands (Fig. 2A). The average molar mass is sufficient to prevent passage through dialysis membrane and DOTAGA functionalization offers the capacity to efficiently chelate excess free (or loosely chelated) iron, copper or zinc ions. Dialysis is performed in recirculation conditions in order to use a small volume of dialysate to avoid strong dilution of important molecules but permits to concentrate the metals by an important factor. Herein, we present the new dialysate formulation concept and we evaluate the safety and the efficacy for endogenous metal extraction on a large animal easily transposable to human: healthy sheep.

## Results

### Characterization and functionalization of chitosan

Functionalized chitosan (Chitosan@DOTAGA) has been synthesized by a two-steps process. The first step consisted in the reacetylation of initial chitosan by addition of pure acetic anhydride in a water/1,2-propanediol mixture^25^. The degree of acetylation is determined by ^1^H NMR and confirmed by IR spectrometry and is close to 30 ± 2% (x = 0.3) (See Supporting Information Figs. S1, S2). The second step of the synthesis is the functionalization of acetylated chitosan by DOTAGA anhydride (1,4,7,10-tetra-azacyclododecane-1-glutaric anhydride-4,7,10-triacetic acid) that is directly added to the precedent solution of reacetylated chitosan in water/1,2-propane-diol. Then, purification was performed by tangential filtration (cut-off: 100 kDa) and the resulting modified chitosan was freeze-dried. Purity (98%) of the product was assessed by SEC (size exclusion chromatography) coupled with UltraViolet–Visible detection (Fig. 2B). A retention time of 6.7 min is observed for polymer (Chitosan@DOTAGA) and a minor peak at 11.3 min is observed for ungrafted DOTAGA.

The efficiency of functionalization was assessed by dosage, after addition of small amounts of Cu(II) on a DOTAGA-modified chitosan solution at 1 g L^−1^. For each addition of copper, UV absorbance at 295 nm was plotted using Chitosan@DOTAGA peak area (Fig. 2C). Thus, hypothesizing that copper ions will only interact with DOTAGA moieties in acidic conditions (See Supporting Information Fig. S3 comparing Acetylated chitosan and Chitosan@DOTAGA), for a solution of 1 g L^−1^ of Chitosan@DOTAGA, a molar concentration of 0.35 mM of DOTAGA has been obtained corresponding to y = 0.072 (See Supporting Information). Using ^1^H NMR, x = 0.28 has been determined on the reacetylated chitosan sample (See Supporting Information Fig. S1). The acetylation degree (x) is obtained through the ratio between the area of the peak at 3.1 ppm over the area in the region 2.9–4.1 ppm following the equation:

$$\frac{{Area_{{2.9 - 4.1\;{\text{ppm}}}} }}{{Area_{{2.0\;{\text{ppm}}}} }} = \frac{6}{3x}.$$

The elemental analysis generally confirms the results found by HPLC–UV and ^1^H NMR. The theoretical ratio C/N for chitosan, acetylated chitosan (acetylation degree 28%) and chitosan@DOTAGA is respectively 5.14, 5.54 and 5.46. The experimental results show a C/N ratio of 5.11, 5.96 and 5.47. The slight mismatch for the acetylated chitosan could be explained by the presence of traces of 1,2 propanediol found also in the ^1^H NMR spectrum, which increase the total quantity of C.

### Efficacy of metal extraction in vitro

**Figure 3:**
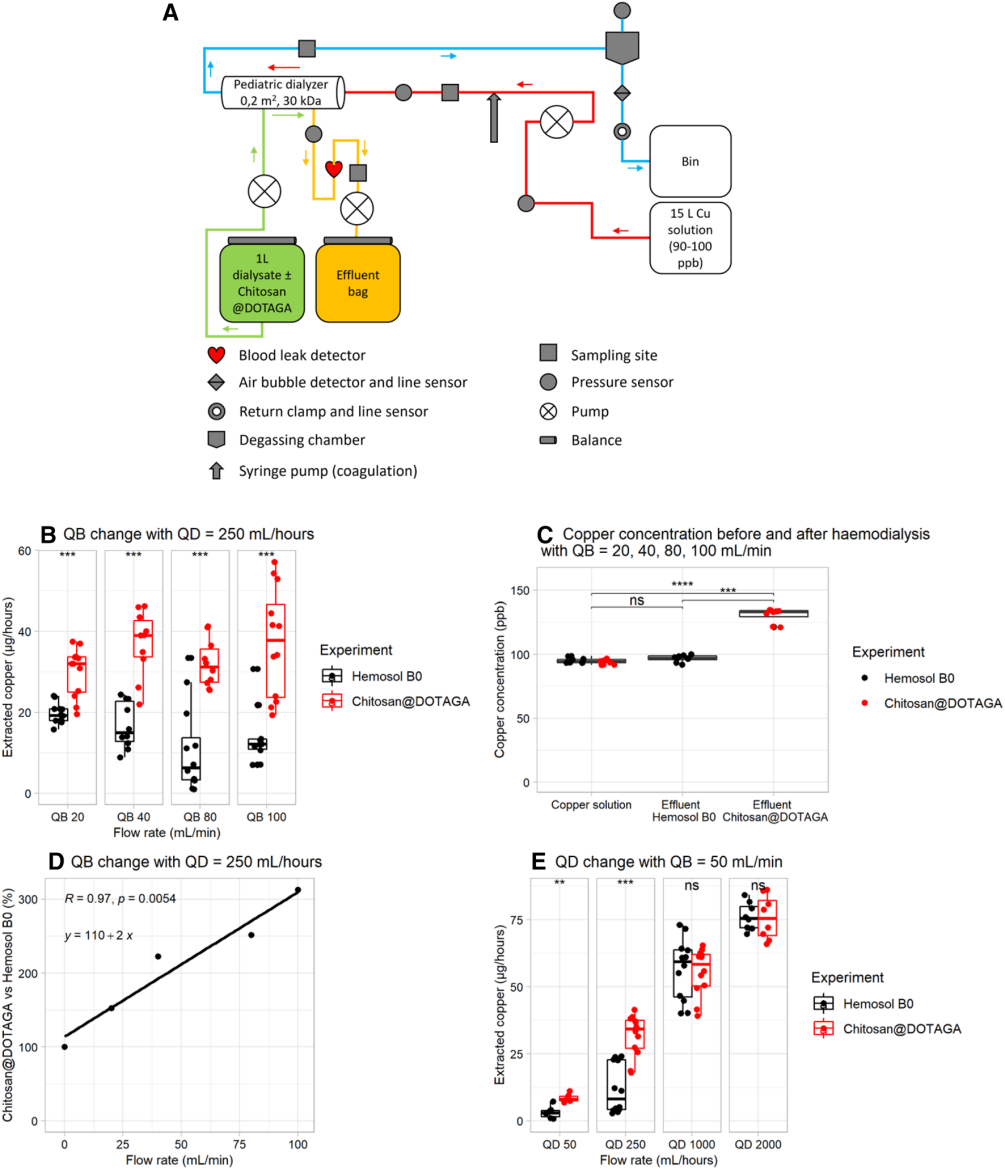
In vitro experiment designated for showing copper extraction due to dialysis with Chitosan@DOTAGA. (**A**) Schematic representation of the experiment. (**B**) Amount of copper extracted (µg/h) from the copper II solution with (red) or without (black) Chitosan@DOTAGA according to increased blood flow rates (QB) (mL/min). (**C**) Copper concentration in the copper solution before dialysis and in the effluent (ppb) with (red) or without (black) Chitosan@DOTAGA with QB = 20, 40, 80 and 100 mL/min and QD (dialysate flow rate) = 250 mL/h. (**D**) Percentage ratio of copper extraction capacity between Chitosan@DOTAGA and the Hemosol B0 alone according to QB (mL/min). The linear regression slope equation ‘y = b + ax’ is presented along with the correlation coefficient (R) and the Pearson correlation *p* value (p). (**E**) Amount of copper extracted (µg/h) from the copper solution with (red) or without (black) Chitosan@DOTAGA according to QD (mL/h). Stars indicates significant differences according to non-parametric Kruskal–Wallis tests (NS: non-significant, *: *p* value < 0.05, **: *p* value < 0.01, ***: *p* value < 0.001).

Copper ions extraction efficacy was first tested in vitro using a HF20 dialyzer with intermediate cut-off membrane (~ 10–20 kDa) in a hemodialysis protocol (Fig. 3A). The copper II solution was placed in the blood compartment at a concentration in copper of 100 ppb and different blood flow rates were tested (QB). In the dialysate compartment, Hemosol B0 alone was compared to Chitosan@DOTAGA at a concentration of 1 g L^−1^ in Hemosol B0 and a fixed dialysate flow rate (QD) of 250 mL/h was used. For each tested flow rate, copper extracted per hour was significantly higher (*p* value < 0.001) in presence of Chitosan@DOTAGA (Fig. 3B). Median values for copper extracted per hour varied respectively from 29 to 38 µg/h and from 6 to 19 µg/h, in presence and in absence of Chitosan@DOTAGA. Copper extraction was confirmed by analysis of copper content in the effluent that is significantly higher for Chitosan@DOTAGA in comparison with Hemosol B0 (*p* value < 0.001) (Fig. 3C). The percentage ratio of copper extraction capacity between Chitosan@DOTAGA and the Hemosol B0 alone was plotted versus flow rate and lead to a linear increase (Fig. 3D), no plateau effect could be reached in the investigated flow rate range. Varying dialysate flow rate while maintaining blood flow rate constant (QB = 50 mL min^−1^) was also tested (Fig. 3E). The presence of Chitosan@DOTAGA in Hemosol B0 leads to an increased extraction of copper at low and moderate QD (*p* value < 0.01 for QD = 50 mL/h and *p* value < 0.001 for QD = 250 mL/h) but no statistical difference is observed at high QD.

### Feasibility and metallic extraction on healthy sheep

**Figure 4:**
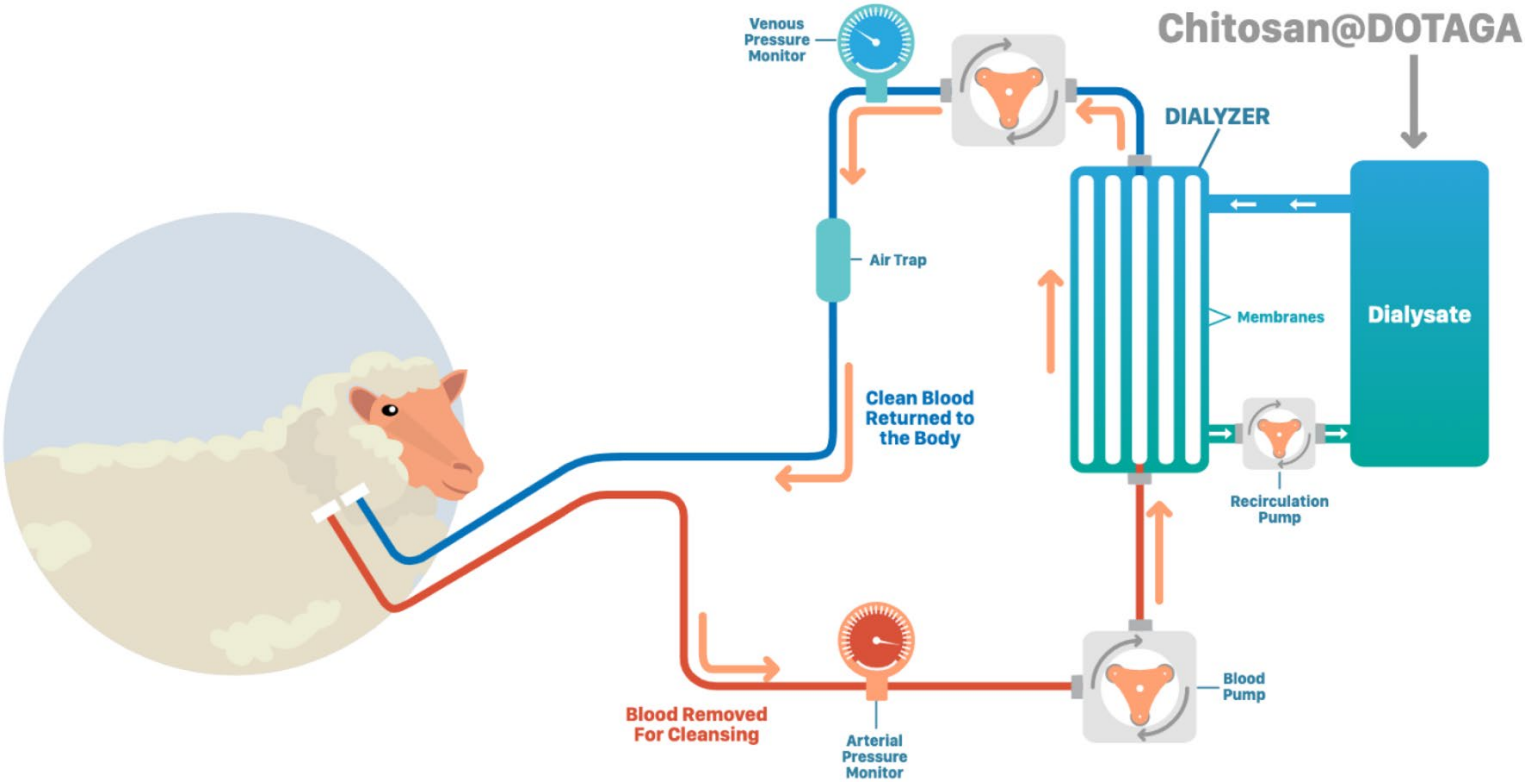
Schematic representation of the metallic extraction experiment on sheep using hemodialysis in recirculation conditions in presence of Chitosan@DOTAGA. Blood is purified by passage in dialysis membrane and metals are retained in the dialysate due to presence of Chitosan@DOTAGA. Two high cut-off membranes (Theralite and SepteX) are compared with standard dialyzer (POLYFLUX H6).

Prior to evaluation on animals, compatibility of Chitosan@DOTAGA with dialyzers membranes has been tested and integrity of membrane has been verified by Scanning Electron Microscopy (SEM) (See Supporting Information Fig. S5). After 4 h of dialysis with Hemosol B0 and Chitosan@DOTAGA on blood, no alteration of the membrane was observed. Further, hemodialysis has been performed on 4 sheep combining different dialyzers membranes and Chitosan@DOTAGA to evaluate the impact of the membrane on safety and metallic extraction efficacy (Fig. 4).

For these experiments, two high cut-off (HCO, 45–60 kDa) membranes (Theralite and SepteX) have been compared with standard dialyzer (POLYFLUX H6, 15–20 kDa cut-off membrane). The protocol of the experiment is described in Table 1. All sheep were submitted to a first hemodialysis treatment with Theralite and a second treatment either with Septex or Polyflux H6.

**Table 1:** Description of in vivo experiments on healthy sheep with different dialyzers membranes.

	Day 0	Day 1 Sheep A and B	Day 2 Sheep C and D	Day 3 Sheep A and B	Day 4 Sheep C and D	Day 7	Day 10
Procedures	Dual lumen catheter placement under anesthesia	Session of HD with Theralite		Session of HD with POLYFLUX H6	Session of HD with SepteX		
Dialysate flow rate (ml/min)		100	100	100	100		
Blood flow rate (ml/min)		100	100	100	100		
Blood tests	1 Before surgery	2 Before and after session		2 Before and after session		1	1

**Figure 5:**
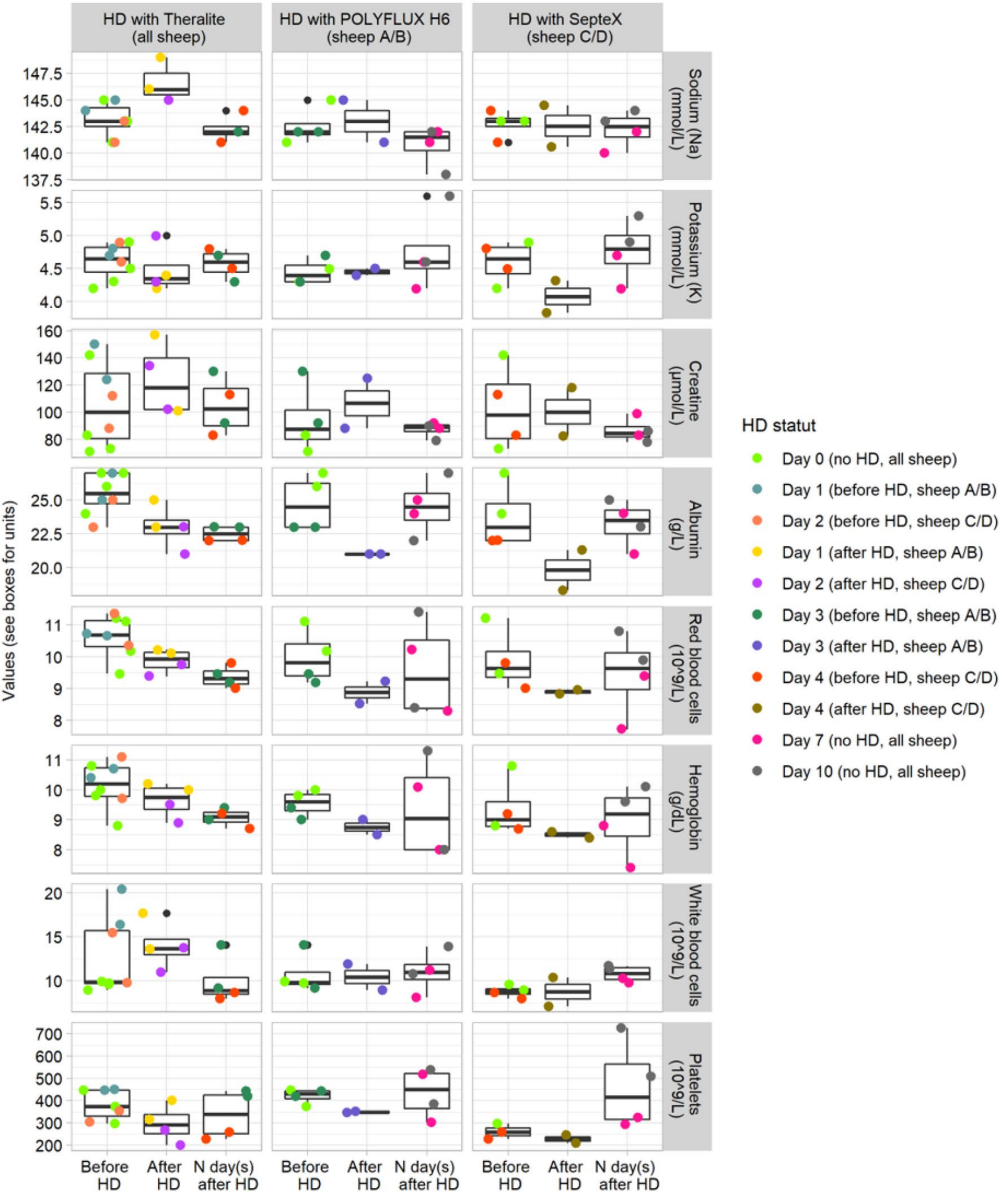
Evolution of a subset of blood parameters according to the experimental and statistical design. Values “before HD” were melted with D0 values to consider the variability within sheep (See column HD with Theralite) or to mitigate the effect of the Theralite HD session in the analysis of both POLYFLUX H6 (sheep A/B, DAY 3) and SepteX (sheep C/D, DAY 4) HD sessions. Note that “before HD” samples of both POLYFLUX H6 and SepteX HD sessions were taken after HD with Theralite and one full resting day for all sheep. Values “after HD” correspond, for all membranes, to the sampling realised at the end of each corresponding HD session. Values “N day(s) after HD” correspond, for the Theralite membrane, to the “before HD” samples of both POLYFLUX H6 and SepteX HD sessions (one full resting day); and to the “DAY 7” and “DAY 10” samples for both POLYFLUX H6 and SepteX membranes (4 to 7 full resting day). The existence of a significant change in abundance with samples categories (before HD, after HD, N day(s) after HD) was tested with a non-parametric Kruskal–Wallis tests. Only the sodium, the albumin and the red blood cells were significantly different for the HD with Theralite membrane (*p* value < 0.05).

Hemodialysis (HD) was performed in dialysate recirculation conditions with sessions of 4 h and a volume of dialysate of 1 L (See Supporting Information Fig. S6). This protocol was applied to limit the diffusion process of blood components while maintaining the blood copper depletion thanks to copper sequestration in the Chitosan@DOTAGA polymer. The dialysis protocol was safe, each animal presented normal behaviors and stayed in healthy conditions post-dialysis (feed, drink, rumination and mobility). Blood analysis (n = 28) were performed one day before the first dialysis session (DAY 0), before and after each dialysis session and 4 (DAY 7) and 7 (DAY 10) days after the last dialysis session. The summary of the full blood analysis (cell blood count, liver enzyme and inflammation) is presented in Supplementary Tables S1 to S3. The evolution of blood parameters according to the samples type (before HD, after HD, N day(s) after HD) per dialysis membranes (Theralite, POLYFLUX H6, SepteX) have been tested by non-parametric Kruskal–Wallis tests, followed by Dunn tests as *post-hoc* when significant. Means, standard deviation and percentages of deviation from the “before HemoDialysis (HD)” samples were also computed for both “after HD” and “N day(s) after HD” samples. Figure 5 displays the results for a subset of blood parameters. No significant differences were observed for the POLYFLUX H6 and SepteX membranes on this subset of data while sodium (mmol/L; *p* value = 0.034 for “after HD” versus “N day(s) after HD”), albumin (g/L; *p* value = 0.023 for “before HD” versus “N day(s) after HD”) and red blood cells (10^12^/L; *p* value = 0.012 for “before HD” versus “N day(s) after HD”) undergoes a significant but small change with the Theralite membrane. Total proteins (g/L; *p* value = 0.034 for “before HD” versus “N day(s) after HD” and *p* value = 0.025 for “before HD” versus “after HD”) and red blood cells distribution width (%;*p* value = 0.049 for “before HD” versus “N day(s) after HD”) also undergoes a small significant change with the Theralite membrane. With the POLYFLUX H6 membrane, the gamma glutamyl transferase (UI/L; *p* value = 0.024 for “before HD” versus “N day(s) after HD”) and the monocytes (10^9^/L: *p* value = 0.04 for “before HD” versus “after HD”) undergo a significant change, finally, with the SepteX membrane, only the gamma glutamyl transferase (UI/L; *p* value = 0.038 for “before HD” versus “N day(s) after HD”) undergoes a significant change. Differences in the number and nature of significantly shifted parameters with the Theralite membrane compared to the POLYFLUX H6 and the SepteX membrane can be explained accounting several aspects (i) the “N day(s) after HD” samples were taken after only 1 resting day for the Theralite membrane in comparison with 4–7 days for both POLYFLUX H6 and SepteX membranes (e.g., albumin, total protein, red blood cells) (ii) the number of available data was higher with the Theralite membrane allowing a slightly better statistical treatment (e.g., sodium, Red blood cells distribution width: no more than 5% of deviation compared to “before HD”, see Supplementary Table S2) (iii) the surface of the Theralite membrane is higher compared to both POLYFLUX H6 and SepteX membranes (2.1 m^2^ versus 0.2 m^2^ and 1.1 m^2^) which favors the diffusion process. Indeed, when looking at the percentage of deviation from the “before HD” samples, both albumin and total protein decreased by 10 to 16% for all membranes in the “after HD” samples. After one resting day, no restauration in albumin and total protein can be observed with the Theralite membrane while after 4 to 7 days, both albumin and total protein were decreased by only 1 to 5% compared to “before HD” samples with both POLYFLUX H6 and SepteX membrane. The same observation was made with the red blood cells with a decrease of 7 to 11% recorded for all membranes in the “after dialysis” samples, no restauration observed after one resting day with the Theralite membrane, and no more than 4.5% decreased after 4 to 7 days in comparison with the “before HD” samples for both POLYFLUX H6 and SepteX membranes. The moderate decreased in red blood cells can be explained by residual volume of blood in haemodialyser lost on return, and via hemolysis which is frequent for patients treated with HD; it was accompanied with non-significant (but consistent across membrane) decreased of hemoglobin (5–8%) and hematocrit (8%). Loss of albumin (and thus total protein by passage through the membrane) was expected with both HCO membranes while not with POLYFLUX H6 membrane. It can be noted that 16% of albumin variation correspond to a change of only 4 g/L with a “before HD” value ranging from 23.8 to 25.5 g/L. According to Morgera et al.^26^ who observed an increase of albumin loss when comparing 1000 mL/h to 2500 mL/h flow rate (from 410 to 950 mg/h which correspond to 1.6 to 3.8 g in 4 h), we can assume that the recirculation protocol limits the loss of albumin with HCO membranes (experimental dialysate flow rate = 6000 mL/h).

**Figure 6:**
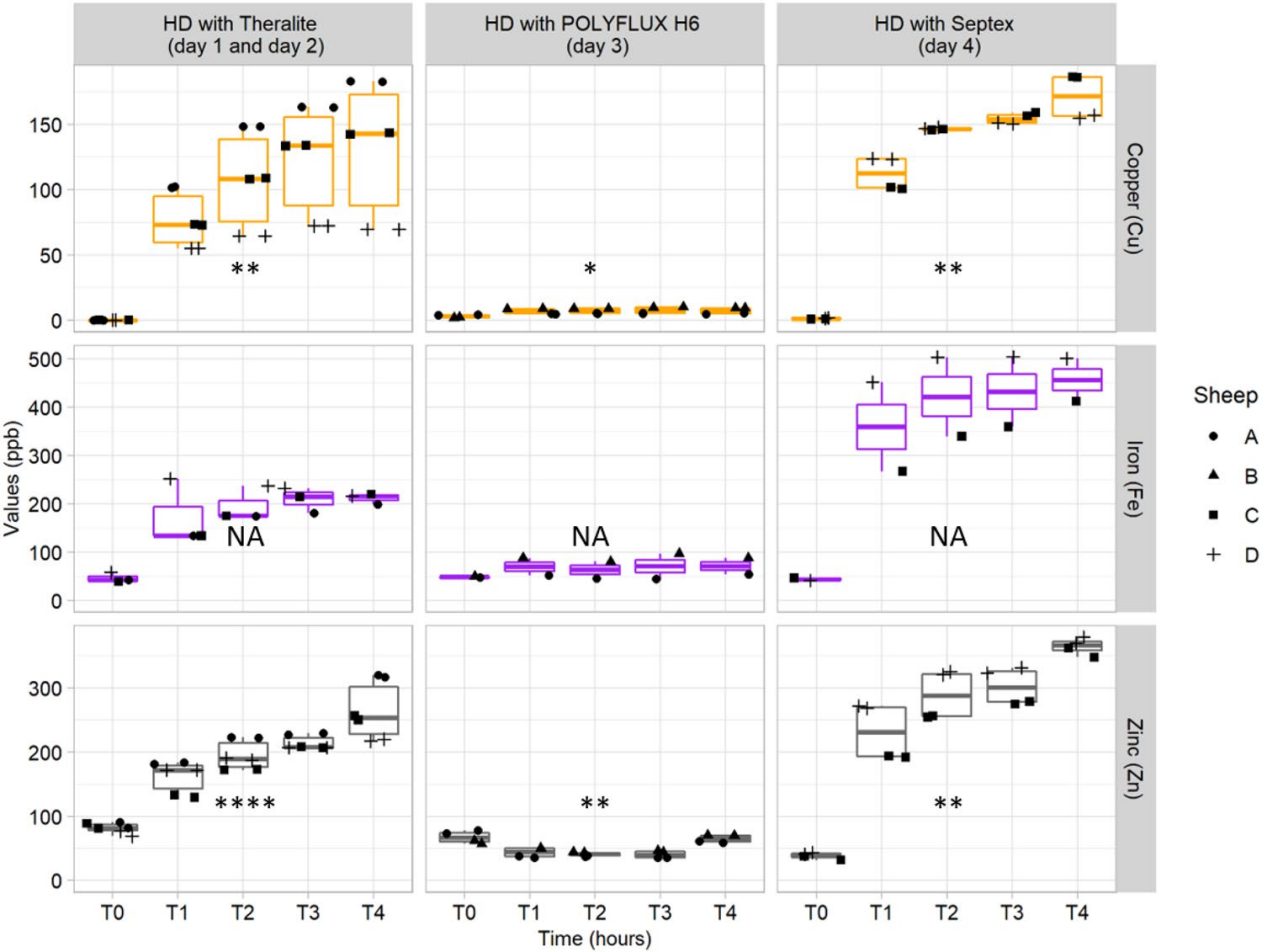
Copper, iron, and zinc (ppb) monitoring by ICP/MS in the dialysis bags along 4 h dialysis sessions on healthy sheep using Theralite membrane (HCO, day 1 and 2, sheep A, C, D), POLYFLUX H6 membrane (HF, day 3, sheep A and B) or SepteX membrane (HCO, day 4, sheep C and D). All sheep (n = 4) experienced two dialysis treatments with one treatment failure, resulting in 7 dialysis sessions only. For copper and zinc, two isotopes were analyzed (Cu 63 and Cu 65; Zn 66 and Zn 68) while iron was only represented by the Fe 56 isotope. Stars indicates significant change in abundance with time according to non-parametric Kruskal–Wallis tests (significance: NS = *p* value > 0.05, * = *p* value < 0.05, ** = *p* value < 0.01, *** = *p* value < 0.001, **** = *p* value < 0.0001, NA = not applicable, insufficient number of measurements to assess significance).

Metallic extraction has been assessed for endogenous metallic cations (iron, copper and zinc) by measuring their quantities by ICP-MS in dialysate, but also in whole blood and plasma for the three different types of dialyzers membranes (POLYFLUX H6, Theralite and SepteX). In dialysate, samples were taken before dialysis, after 1, 2, 3 and 4 h of dialysis (Fig. 6 and Table S4). For the three metals (Table S5), HCO membranes presented the same behavior with clear and important increase of the quantity of metal with time. In terms of efficacy, SepteX membrane can extract close to 0.2 mg of copper and 0.5 mg of iron on a healthy animal, in 4 h. On the other hand, POLYFLUX H6 membrane leads to negligible extraction for zinc and relatively small one for iron and copper. These results have been compared with metal analysis in total blood (See Supporting Information Fig. S6) and in plasma (See Supporting Information Fig. S7). For iron, only plasma measurements have been taken due to too large quantities of iron in blood cells. These trends are coherent with metallome study in dialysate especially for SepteX where an important decrease of metallic level is observed in plasma (See Supporting Information Fig. S8).

## Discussion and conclusion

Extraction of metals is a requirement for pathologies where excess of metals is recognized to be one of the hallmarks of the pathology like Wilson disease or primary and secondary hemochromatosis. For these pathologies, the current treatments include copper chelators for Wilson disease or simple phlebotomy or iron chelators for hemochromatosis. These treatments are relatively well adapted for chronic pathology but cannot avoid neurological worsening for certain patients in Wilson disease and are also not adapted for rapid metal purification for patients that suffer burst of free metal liberation as it can occur in intensive care units due to secondary effects occurring at high concentration of chelates. On the other hand, dialysis is already currently used in intensive care units. Developing a medical device for rapid extraction of metals using dialysis can be easily adapted in the medical practice for those patients. Combining dialysis with chelating polymer is a new concept that is based on the chelation in the dialysate of free metals that leads to an overconcentration of metals in the dialysate that cannot go back to the blood circulation due to the size of the polymer and the cut-off of the membrane. It permits to have effective concentration of metals with recirculation avoiding using large volume of dialysate.

Chitosan has been chosen as a functional macrochelatant for this application because (i) it can be easily functionalized on the primary amines moieties, (ii) it is biocompatible, (iii) it is soluble at physiological pH after partial reacetylation (DA ~ 30%) and DOTAGA grafting, (iv) it can exhibit high molecular mass, sufficient not to pass through dialysis membrane and (v) it is affordable as clinical grade and can be obtained at large scale. DOTAGA (1,4,7,10-tetraazacyclododecane-1,4,7,10-tetraacetic acid) has been chosen as chelate for this application due to its high complexation constants for Cu^2+^, Fe^3+^ and Zn^2+^ (log β = 22.44, 29.4 and 20.52 respectively)^27^. For the functionalization on chitosan, DOTAGA anhydride has been chosen due to its additional reactive site to avoid losing a carboxylic acid group implicated in the coordination of metals^28,29^. In our conditions, Chitosan@DOTAGA displays high solubility at 1 g L^−1^ and can chelate 0.35 mM of metals thus leading to a maximum extraction capacity of 19.5 mg of iron or 22.2 mg of copper per liter of dialysate solution.

It has been shown in vitro that using standard dialyzer HF20, important copper extraction can be reached at moderate dialysate flow rate by increasing blood flow rate. A feasibility study has been performed on healthy sheep comparing 3 different types of dialyzers membrane: POLYFLUX H6, SepteX and Theralite (i.e. one standard dialyzer and two high cut-off membranes) and recirculation conditions for 1 L of dialysate. Sheep have been chosen, as large animal model, for future translation due to their easy taking care in dialysis experiments. Interestingly, no secondary effect has been observed on the sheep with no significant modifications of hematological, immunological or liver enzymatic parameters and the sheep are still living 12 months after the experimental protocol. During dialysis sessions of 4 h with classical POLYFLUX H6 dialyzer membranes, we found a modest extraction of 10–50 µg of free iron and free copper. Using HCO membranes, about ten times higher uptakes have been evidenced with about 200 and 500 µg copper and iron extracted respectively on healthy sheep that present only small quantities of free metals in comparison with diseased ones, that is encouraging for future application like Wilson disease and further clinical translation.

## Material and methods

### Synthesis of Chitosan@DOTAGA

#### Chemicals

Medical grade chitosan of low degree of acetylation (DA) from Alaska snow crab was purchased from Matexcel (Bohemia, NY, USA, reference number NAT-0030, https://www.matexcel.com/p/30/medical-grade-chitosan/). The weight-average and number-average molar masses (respectively Mw = 2.583 10^5^ g/mol, Mn = 1.323 10^5^ g/mol) were determined by size exclusion chromatography coupled with refractive index and multi-angle laser light scattering measurements^30^. The initial degree of acetylation of such raw chitosan was determined by ^1^H NMR spectroscopy by the Hirai method^31^ close to 4.5 ± 0.5%. DOTAGA anhydride has been furnished by CheMatech (Dijon, France). 1,2-propanediol and acetic anhydride were furnished by Sigma-Aldrich (France). Acetic acid was furnished by VWR (France).

#### Acetylation of chitosan

60 g of chitosan (258 kDa) are poured in a 10 L reactor then 50 mL of acetic acid and 4 L of milliQ water are added and the solution is stirred for 16 h. 1.2 L of 1,2-propanediol are then added and the solution is stirred at room temperature for 1 h. Then 14 mL of acetic anhydride dissolved in 600 mL of 1,2-propanediol are slowly added (10 min) for homogeneous acetylation. The mixture is stirred for 4 h. Some of the product is extracted and purified by tangential filtration with water for characterization of the intermediate product.

#### Functionalization of acetylated chitosan

120 g of DOTAGA anhydride are added to 2 L of the precedent solution and the mixture is stirred for 16 h. Then, the solution is diluted by a factor 10 in milli Q water and purified by tangential filtration using a 100 kDa membrane (Sartocon® Slice 200, PES membrane) with a Sartoflow® Smart apparatus. Tangential filtration is used for the two other purification steps towards acetic acid at 0.1 M and milli Q water.

#### Characterization by HPLC–UV using SEC columns

HPLC-SEC-UV chromatograms were recorded with an Agilent-1200 HPLC apparatus with a DAD module. The SEC column used is a PolySep-GFC-P 4000 and a 0.1 M solution of acetic acid and 0.1 M ammonium acetate is used as a buffer. The operating temperature is 30° C and the absorption wavelength is 295 nm. The eluent flow rate is 0.8 mL/min.

#### Infrared spectroscopy

Fourier-transform infrared spectroscopy spectra were recorded using a Shimadzu IRAffinity-1 equipped with a DLaTGS detector, using the lyophilized powder as samples and recording in a range between 400 and 4000 cm^−1^ with 32 scans and a resolution of 0.5 cm^−1^.

#### Nuclear magnetic resonance

The NMR ^1^H is performed at the CCRMN Lyon platform on a Bruker 400 MHz and the spectra are analyzed with the software Mestrenova (Version: 6.0.2-5475. 2009 Mestrelab Research S. L.).

#### Elemental analysis

The analysis is made by the “Pôle Isotopes & Organique” of the Institut des Sciences Analytiques, UMR 5280.

### In vitro efficacy of copper extraction by chelating dialysis

#### Chemicals

The standard Cu solution for the ICP-MS is provided by SCP-Science: ICP Standard Cu 50,000 ug/mL 140-041-295 SCP Science. The dialysate solution used for the experiment is the Hemosol B0 produced by Baxter. The other chemicals used for the preparation of the copper II solution is the sodium chloride (purity > 99.8%, CAS 7647-14-5) provided by Carl Roth, the sodium hydrogen carbonate (analytical reagent grade, CAS 144-55-8) provided by Fisher Chemical and the citric acid (ACS reagent > 99.5%, CAS 77-92-9) provided by Merck.

#### Preparation of copper solution

15 L of copper solution are prepared for each experiment with and without Chitosan@DOTAGA. The 15 L of solutions contain 0.8 mM of citric acid, 35 mM of sodium bicarbonate, 105.3 mM of sodium chloride and 100 ppb of copper II.

#### Preparation of Chitosan@DOTAGA and Hemosol B0 only dialysates

1 L or 900 mL of Hemosol B0 were sampled manually from the 5 L reconstituted Hemosol B0 solution and introduced in a clean dialysis bag. 1 L of Hemosol B0 were used to conduct the experiment without Chitosan@DOTAGA and 900 mL of Hemosol B0 were completed with 150 mL of Chitosan@DOTAGA (7 g/L) to obtain a concentration of 1 g/L of Chitosan@DOTAGA.

#### Experimental protocol

The dialysis protocol is summarized in Fig. 3A using a Prismaflex monitor and a HF20 set (0.2 m^2^) as dialyzer.

First, the dialysis flow rate is maintained constant at 250 mL h^−1^ and 4 blood rates are tested for 30 min each (20 mL min^−1^, 40 mL min^−1^, 80 mL min^−1^ and 100 mL min^−1^). For each flow rate, sampling of the Cu solution in duplicate at T0 (red sampling site), T10 min, T20 min and T30 min (blue sampling site). Sampling of the dialysate/effluent at T0 (Chitosan@DOTAGA only) in the dialysis bag and T20 min (yellow sampling site).

Then, the blood flow rate is maintained constant at 50 mL min^−1^ and 4 dialysate flow rates have been tested: (i) 5 min at 2000 mL/h: sampling of the Cu solution at T0 (red sampling site), T2.5 min, T5 min (in duplicate, blue sampling site); sampling of the effluent at T2.5 min (yellow sampling site), (ii) 7.5 min at 1000 mL/h: sampling of the Cu solution at T2.5 min, T5 min, T7.5 min (in duplicate, blue sampling site); sampling of the effluent at T5 min (yellow sampling site), (iii) 30 min at 250 mL/h: sampling of the Cu solution at T10 min, T20 min, T30 min (in duplicate, blue sampling site); sampling of the effluent at T20 min (yellow sampling site) and (iv) 60 min at 50 mL/h: sampling of the Cu solution at T0, T30 min, T60 min (in duplicate, blue sampling site); sampling of the effluent at T30 min (yellow sampling site). Both experiments were conducted with and without Chitosan@DOTAGA.

#### Copper quantification by ICP-MS

ICP-MS analysis were performed using a Perkin Elmer NexION2000 equipped with Syngistix software (Licensed software by Perkin Elmer. Version: 2.3 (Build 2.3.7916.0)) and a ESI SC-FAST Sample Introduction in Kinetic Energy Discrimination (KED) mode. The samples are prepared by dilution in an aqueous solution of HNO_3_ 1% v/v.

### In vivo proof of concept on healthy sheep

This study was conducted in accordance with the Guide for the Care and Use of Laboratory Animals and approved by the Ethics Committee of VetAgroSup (Campus vétérinaire de Lyon) with the agreement 1548-V2. The study was carried out in compliance with the ARRIVE guidelines.

#### Animals

4 adult male sheep weighing 44 to 56 kg were included in this study. 14-day acclimation period was implemented before the study. Animals were fed with hay ad libitum and with alfalfa pellets and given free access to water. The day before the experiment each sheep was equipped under anesthesia (intramuscular injection of xylazine (0.1 mg/kg) and midazolam (0.2 mg/kg)) with a 11.5Fr double lumen catheter (Hemo-cath®, Medical Components, Harleysville, PA) inserted in the right jugular vein with the transcutaneous Seldinger technique (Pouzot-Nevoret and al, 2017). The day of the hemodialysis, two sheep were installed together in the dialysis room to respect animal welfare. At the end of the day, the animals were placed in facility in a common box. The physiological status pre, per and post dialysis session of each sheep was evaluated through the quantification of the food and drink ingested, and the observation of rumination mobility and behavior.

#### Hemodialysis protocol

The dialysis is performed in recirculation using the cascade dialysis prototype from Baxter to have manual control over all the parameters during the dialysis. The following parameters have been chosen for the hemodialysis: (i) blood flow rate of 100 mL min^−1^, (ii) dialysate flow rate of 100 mL min^−1^, (iii) dialysate volume of 1 L, (iv) concentration of Chitosan@DOTAGA in the dialysate of 1 g L^−1^ and (v) 4 h of dialysis. The dialysate solution used was Hemosol B0 supplemented with potassium at 4.5 mmol/L.

#### Blood tests

Analysis were performed on KONELAB analyzer (Thermo Fisher Scientific 95615 CERGY PONTOISE France).

#### Metal extraction quantification

Blood samples were collected at day 0, 7 and 10 in the morning and day 1 and 3 before and after dialysis using Vacuette tubes (NH Trace Elements Sodium Heparin 6 mL; Greiner, Kremsmünster, Autriche). ICP-MS analysis were performed using a Perkin Elmer NexION2000 equipped with Syngistix software and a ESI SC-FAST Sample Introduction in Kinetic Energy Discrimination (KED) mode. The samples are prepared by dilution in an aqueous solution of HNO_3_ 1% v/v.

#### Statistics

Both blood analysis evolution according to samples type (“before HD, “after HD”, “N day(s) after HD”) and dialysis bag metals evolution according to time (hours) were tested by non-parametric Kruskal–Wallis tests (krustal.test from the vegan package, Oksanen et al.^32^), followed by Dunn tests (kwAllPairsDunnTest from the PMCMRplus package, Pohlert^33^) for multiples comparison when significant. The ggplot2 package (Wickham^34^) was used for plotting the data along with the stat_compare_mean option (method = “kruskal.test”, package ggpubr, Kassambara^35^) to display statistical differences. All tests were performed on RStudio (v4.0.3, RStudio team^36^).

## Supplementary Information

Supplementary Tables.

Supplementary Figures.
