# Supplementary material for: Feasibility study and direct extraction of endogenous free metallic cations combining hemodialysis and chelating polymer
Source: Sci Rep. 2021 Oct 7;11:19948. doi: 10.1038/s41598-021-99462-y (PMC8497614; doi:10.1038/s41598-021-99462-y)
Supplement: Supplementary file 2 — Supplementary Figures. [file 41598_2021_99462_MOESM2_ESM.docx]

**Feasibility study and direct extraction of endogenous free metallic cations combining hemodialysis and chelating polymer.**

**Marco Natuzzi,^1,2^ Coralie Grange,^1,2^ Thomas Gréa,^2,5^ Thomas Brichart,^1^ Axel Aigle,^1^ Denise Bechet,^1^ Benoit Hautefeuille,^2^ Eloise Thomas,^3^ Jean-Yves Ayoub,^4^ Jeanne-Marie Bonnet,^4^ Vanessa Louzier,^4^ Bernard Allaouchiche,^4^ Aymeric Couturier,^5^ Alexandra Montembault,^6^ Paula Nunes de Oliveira,^6^ Laurent David,^6^ François Lux*,^1,2,7^ Olivier Tillement.^1,2^**

^1^ MexBrain - 305 rue des fours 69270 Fontaines-Saint-Martin.

^2^ Institut Lumière-Matière, UMR 5306, Université Lyon1-CNRS, Université de Lyon, Villeurbanne Cedex, France; Institut Universitaire de France, Paris, France.

^3^ LAGEPP, CNRS UMR 5007, Univ Claude Bernard Lyon 1, Université de Lyon, 69622 Villeurbanne, France.

^4^ APCSe Agressions Pulmonaires et Circulatoires dans le Sepsis, Université de Lyon, VetAgro Sup - Campus Vétérinaire de Lyon, 1 Avenue Bourgelat, 69280, Marcy-l'Étoile, France.

^5^ Service de Néphrologie et Dialyse, Assistance Publique-Hôpitaux de Paris (APHP), Hôpital Universitaire Ambroise Paré, Boulogne Billancourt, France.

^6^ IMP, CNRS UMR 5223, Univ Claude Bernard Lyon 1, Univ Lyon, 15 bd Latarjet, 69622 Villeurbanne, France.

^7^ Institut Universitaire de France (IUF), 75231 Paris, France.

* Correspondence and requests for materials should be addressed to Dr François Lux (email: francois.lux@univ-lyon1.fr)

**Characterization of Chitosan@DOTAGA.**


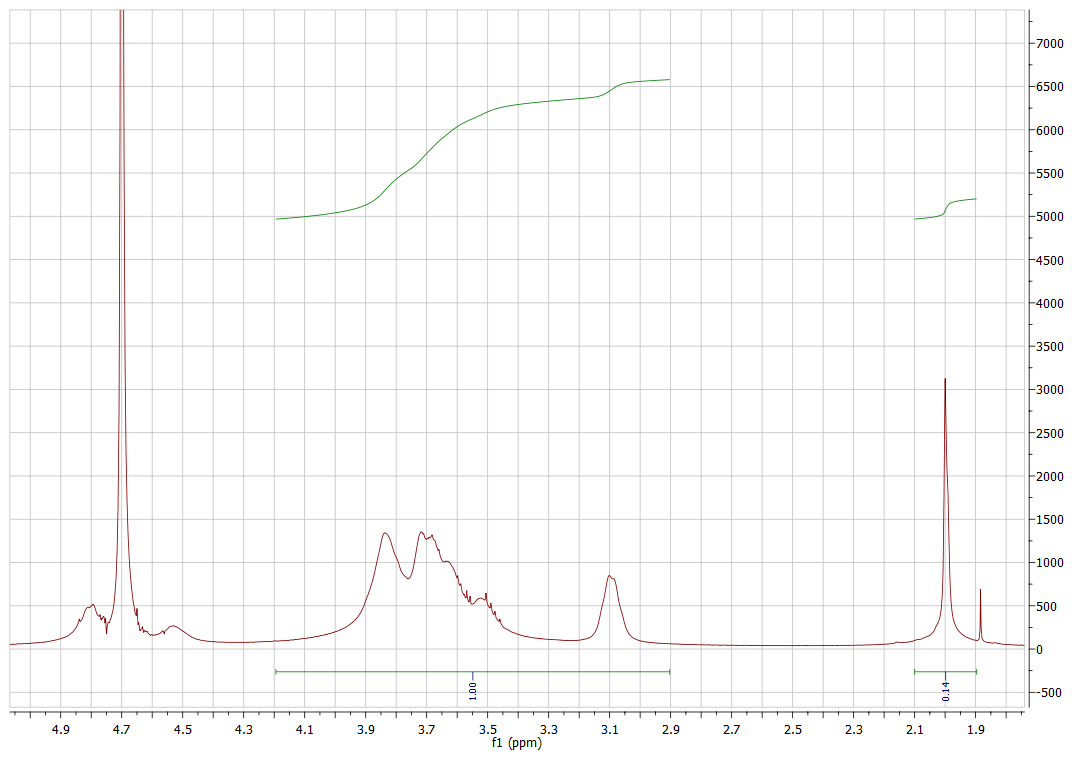


**Figure S1.** RMN spectrum of acetylated chitosan in D_2_O (x = 0.28). Peak close to 2.0 ppm is associated to protons of acetylated group.

**
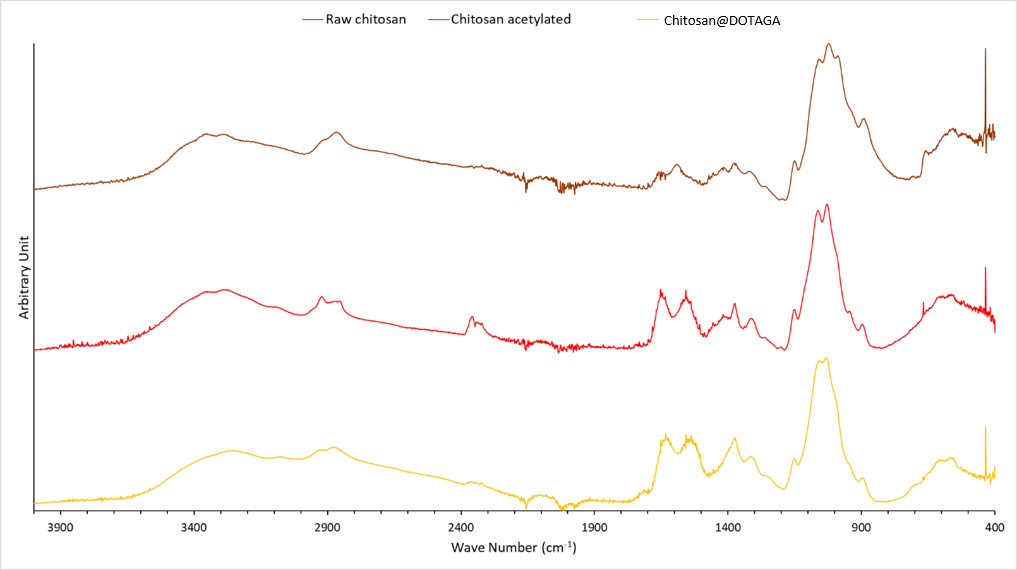
**

**Figure S2.** IR spectrum of raw chitosan, re-acetylated chitosan and Chitosan@DOTAGA


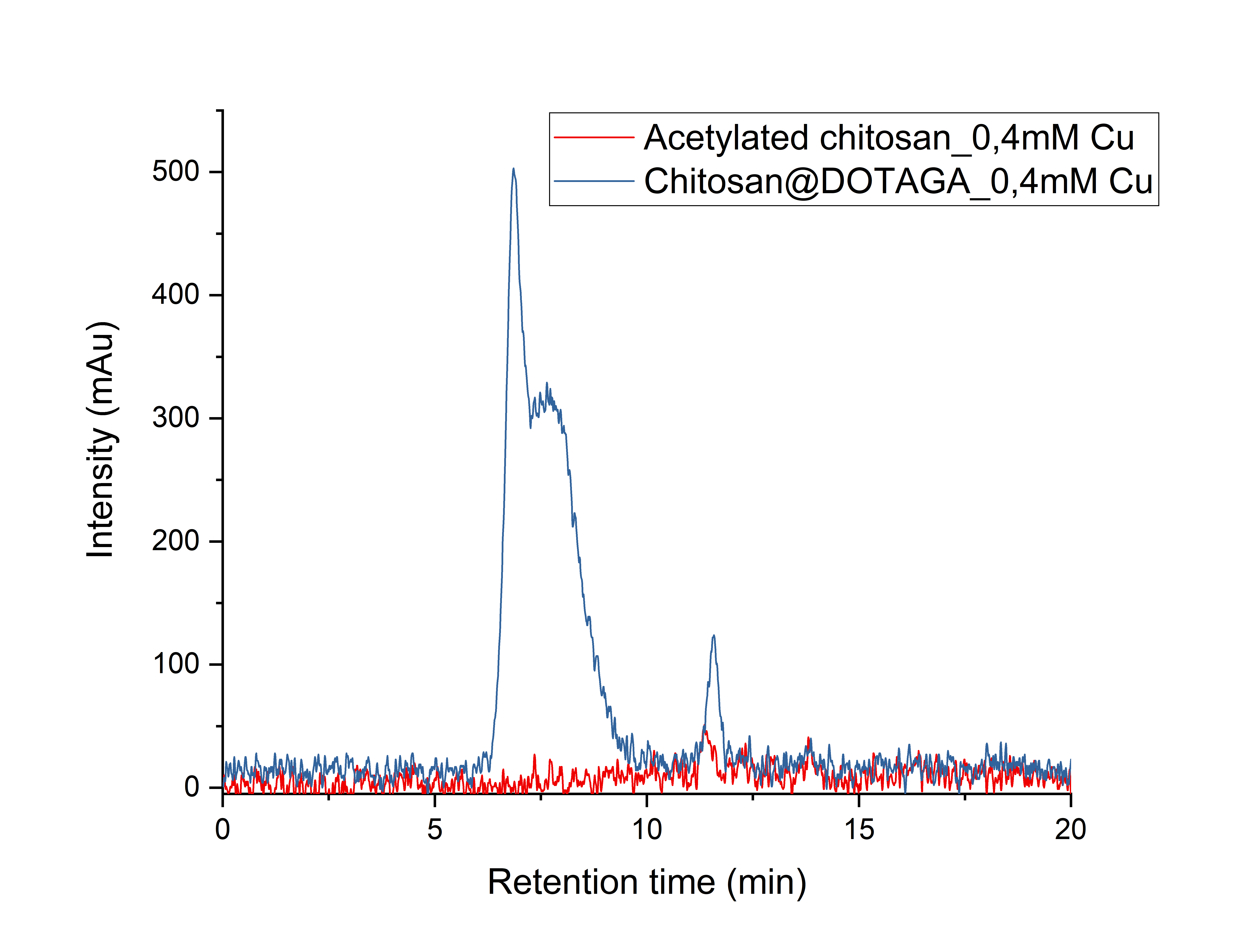
**Figure S3.** HPLC-UV of reacetylated chitosan (x = 0.28) and Chitosan@DOTAGA at 1g/L after addition of Cu (II) 0,4mM. λ_Abs_=295nm. No chelation of copper by reacetylated chitosan is observed by HPLC-UV.

**
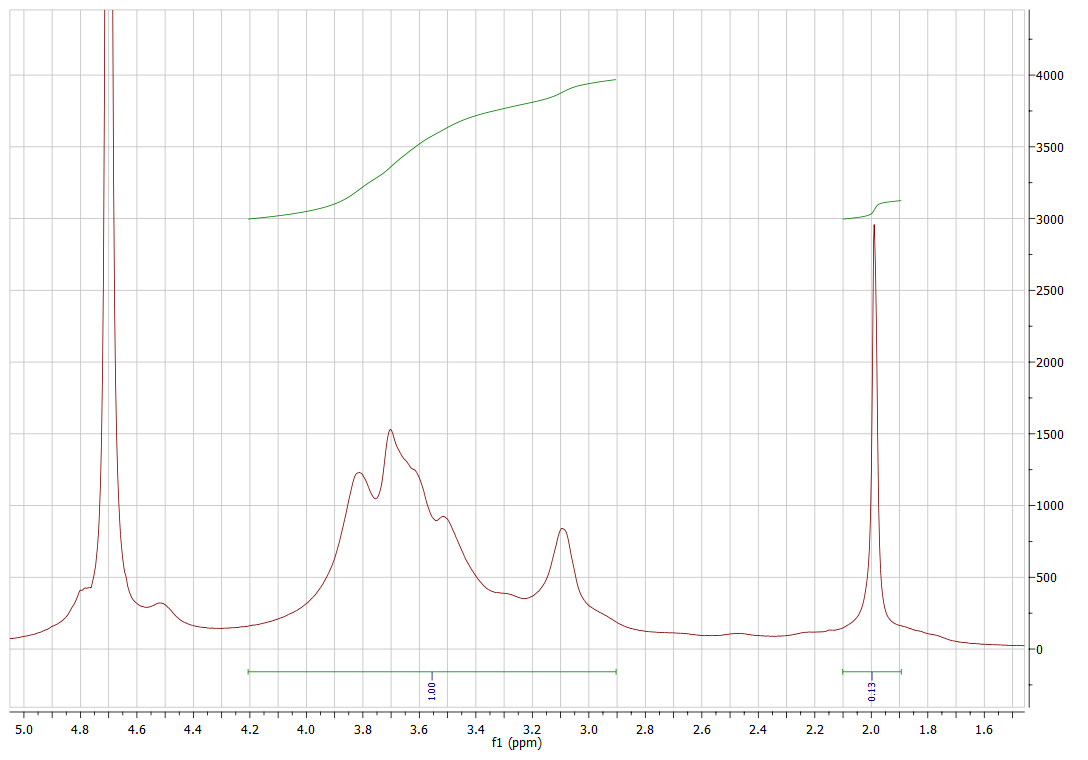
Figure S4.** NMR spectrum of Chitosan@DOTAGA in D_2_O.

**Computation of parameter *y* from HPLC-UV data.**

The mass fraction of the DOTAGA-grafted repeat unit, $w,$ is defined as

$$w\text{ }=\frac{{n_{DOTAGA}\cdot M}_{y}}{m_{Chito}}= \frac{y\cdot M_{y}}{x\cdot M_{x}+y\cdot M_{y}+{z\cdot M}_{z}}$$

Where $n_{DOTAGA}$ is the mole number of DOTAGA-grafted repeat unit, $m_{Chito}$ is the (dry) mass of dissolved chitosan@DOTAGA in the solution, $x$ is the molar fraction of N-acetyl glucosamine repeat units, $y$ =$n_{DOTAGA}/n_{TOT}$ is the molar fraction of the DOTAGA-grafted repeat units and $z$ is the molar fraction of the glucosamine repeat units. Accordingly, $M_{x}$ is defined as the molar mass of N-acetyl repeat units, $M_{y}$ is the molar mass of the DOTAGA-grafted repeat unit and $M_{z}$ is the molar mass of glucosamine residue.

Since $z=1-x-y$ the equation above becomes

$$w=\frac{y\cdot M_{y}}{x\cdot\left( M_{x}-M_{z} \right)+y\cdot\left( M_{y}-M_{z} \right)+M_{z}}$$

Therefore

$$y\text{ }= \frac{w\cdot(x\cdot\left( M_{x}-M_{z} \right)+M_{z})}{M_{y}-w\cdot\left( M_{y}-M_{z} \right)}$$

By computation,

$$M_{x}=203,19\frac{g}{mol} , M_{y}=619,62\frac{g}{mol} , M_{z}=161,16\frac{g}{mol}\text{}$$

Using $m_{TOT}$= 1 g of hydrated chitosan@DOTAGA dissolved in solution, from the HPLC-UV data, we find

$$n_{DOTAGA}=0,335mmol$$

In order to evaluate the mass fraction $w$, we evaluated a water content of $w$c=4.5% w/w in the lyophilized chitosan@DOTAGA powder by a Karl Fischer test. Then,

$$w\text{ }=\frac{{n_{DOTAGA}\cdot M}_{y}}{m_{TOT}\cdot(1-wc)}$$

Hence, $w\text{ }=$0.217

From 1H-NMR,

$$x=0,28$$

This results in

$$y=0,072$$

**In vivo proof of concept on healthy sheep.**

Scanning Electron Microscopy (SEM) was performed with a FEI Quanta 250 FEG instrument. Membranes were deposited on conductive adhesive tabs fixed on flat steel holders before or after dialysis of blood by hemosol or hemosol combined to Chitosan@DOTAGA. Then, samples were coated under vacuum by cathodic sputtering with copper (10 nm) and observed by SEM under an accelerating voltage of 5 kV

**
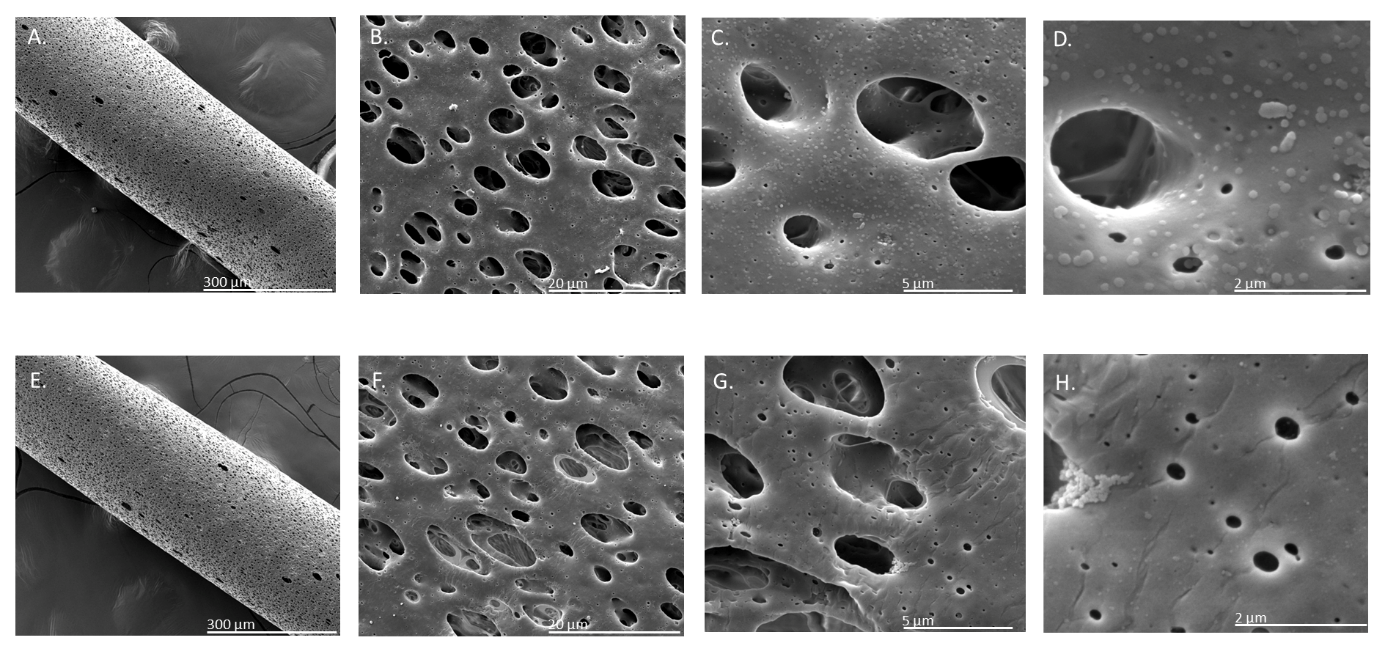
**

**Figure S5.** SEM images of Theralite HCO membrane (Baxter) after dialysis of blood by hemosol (A., B., C., D.) or hemosol combined to Chitosan@DOTAGA (E., F., G., H.).

**
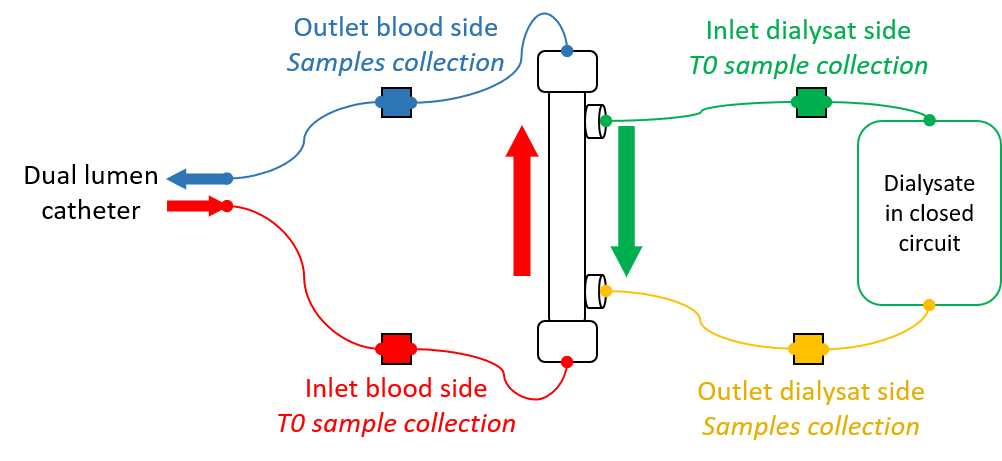
**

**Figure S6.** Schematic representation of the recirculation hemodialysis protocol with sampling sites.


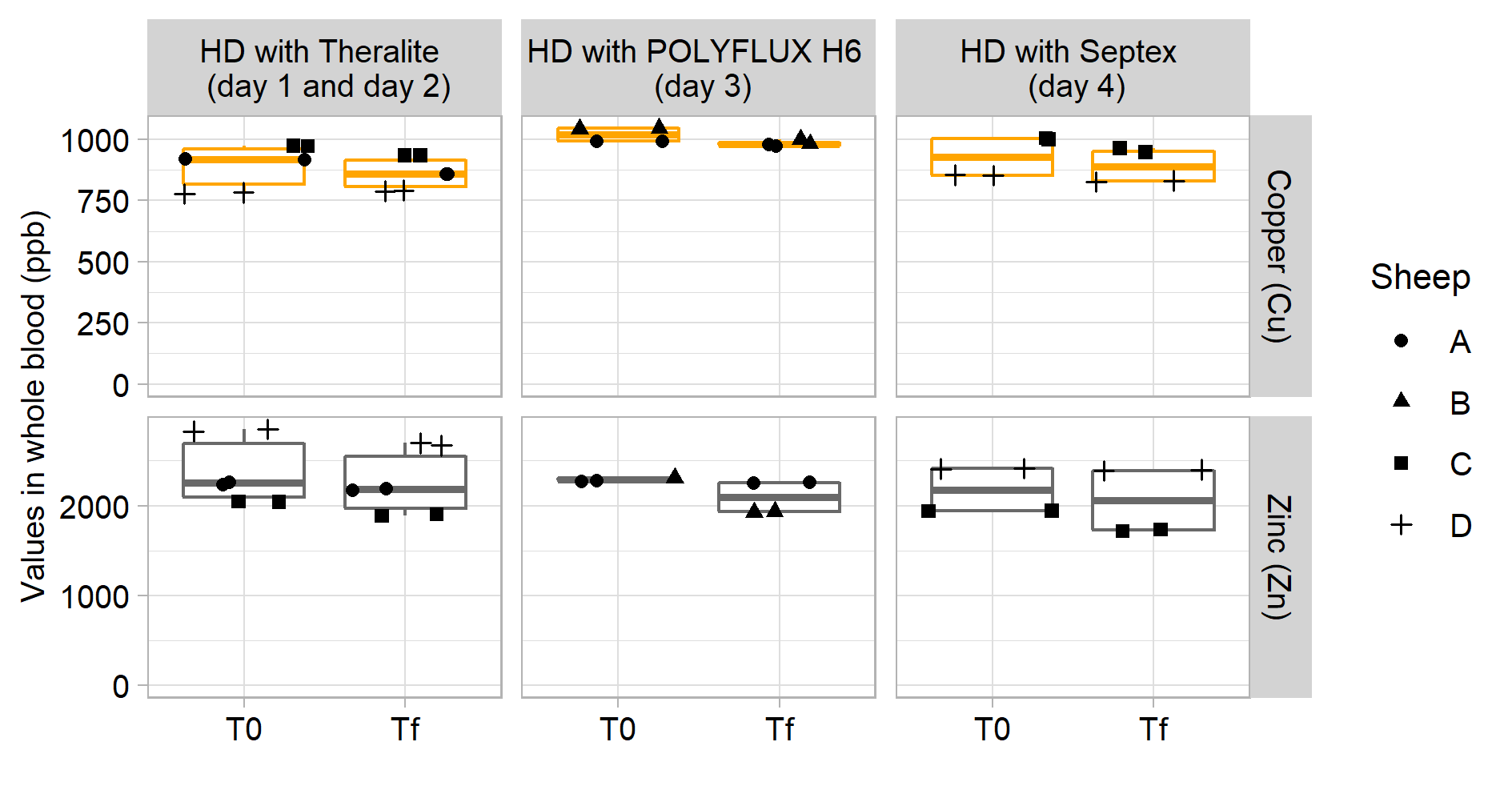


**Figure S7.** Evolution of copper and zinc levels in whole blood before and after dialysis combined with Chitosan@DOTAGA with three different types of dialyser membranes.


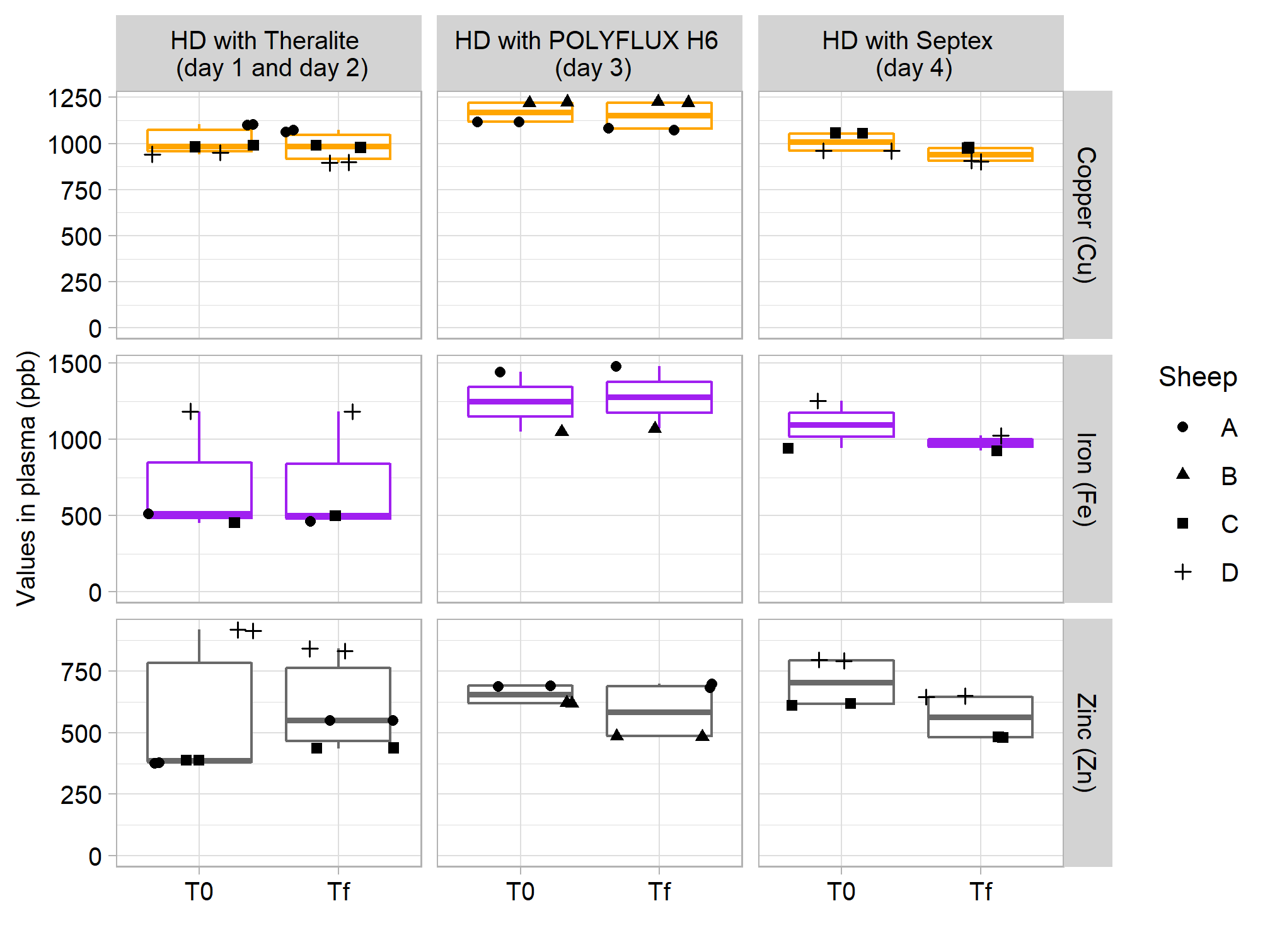


**Figure S8.** Evolution of copper, iron and zinc levels in plasma before and after dialysis combined with Chitosan@DOTAGA with three different types of dialyser membranes.
